# Clinical trial simulation to evaluate tenofovir disoproxil fumarate/emtricitabine HIV pre-exposure prophylaxis dosing during pregnancy

**DOI:** 10.3389/frph.2023.1224580

**Published:** 2023-09-27

**Authors:** Rachel K. Scott, Yifan Yu, Mark A. Marzinke, Jenell S. Coleman, Craig W. Hendrix, Robert Bies

**Affiliations:** Women’s Health Research, MedStar Health Research Institute, Washington, DC, United States; ^2^ Department of Pharmaceutical Sciences, University of Buffalo, Buffalo, NY, United States; ^3^ Division of Clinical Pharmacology, Johns Hopkins University School of Medicine, Baltimore, MD, United States; ^4^ Department of Gynecology and Obstetrics, Johns Hopkins University School of Medicine, Baltimore, MD, United States

**Keywords:** pregnancy, pre-exposure prophylaxis, HIV infection, tenofovir, emtricitabine, clinical trial simulation, population pharmacokinetic modeling

## Abstract

**Objective:**

To evaluate upward-adjustment of tenofovir disoproxil fumarate (TDF)/emtricitabine (FTC) pre-exposure prophylaxis (PrEP) dosing during pregnancy in order to maintain target plasma concentrations associated with HIV protection.

**Design:**

Population pharmacokinetic (PK) modeling and clinical trial simulation (CTS).

**Material and methods:**

We developed population pharmacokinetic models for TFV and FTC using data from the Partners Demonstration Project and a PK study of TDF/FTC among cisgender women by Coleman et al., and performed an in-silico simulation. Pregnancy-trimester was identified as a significant covariate on apparent clearance in the optimized final model. We simulated 1,000 pregnant individuals starting standard daily oral TDF/FTC (300 mg/200 mg) prior to pregnancy. Upon becoming pregnant, simulated patients were split into two study arms: one continuing standard-dose and the other receiving double standard-dose throughout pregnancy.

**Results:**

Standard-dose trough TFV concentrations were significantly lower in pregnancy compared to pre-pregnancy, with 34.0%, 43.8%, and 65.1% of trough plasma concentrations below the lower bound of expected trough concentrations presumed to be the protective threshold in the 1st, 2nd, and 3rd trimesters, respectively. By comparison, in the simulated double-dose group, 10.7%, 14.4%, and 27.8% of trough concentrations fell below the estimated protective thresholds in the 1st, 2nd, and 3rd trimesters, respectively. The FTC trough plasma concentration during pregnancy was also lower than pre-pregnancy, with 45.2% of the steady-state trough concentrations below the estimated protective trough concentrations of FTC. In the pregnancy-adjusted double-dose group, 24.1% of trough plasma concentrations were lower than protective levels.

**Conclusions:**

Our simulation shows >50% of research participants on standard dosing would have 3rd trimester trough plasma TFV concentrations below levels associated with protection. This simulation provides the quantitative basis for the design of prospective TDF/FTC studies during pregnancy to evaluate the safety and appropriateness of pregnancy-adjusted dosing.

## Introduction

Pre-exposure prophylaxis (PrEP) is critically important for the prevention of Human Immunodeficiency Virus (HIV) during pregnancy, both for prevention of maternal HIV and secondary perinatal transmission. Oral tenofovir disoproxil fumarate/emtricitabine (TDF/FTC) is the most commonly used PrEP medication for people with receptive vaginal exposure to HIV and has extensive safety data in pregnancy; however, dosing and efficacy have not been prospectively evaluated in pregnancy. Multiple studies of TDF/FTC during pregnancy both for treatment and prevention of HIV report lower tenofovir (TFV) exposures in the 2nd and 3rd trimesters attributed to pregnancy-related increased volume of distribution and renal clearance (1–16). Similar declines in FTC concentrations are also reported (7–9, 11). The Partners Demonstration Project showed the largest decline during pregnancy compared to non-pregnant women, with 45%–58% reductions in plasma TFV and intraerythrocytic TFV diphosphate (TFV-DP) concentrations from dried blood spots, respectively, compared to non-pregnant women (1). Decreases in peripheral blood mononuclear cell (PBMC) TFV-DP concentrations of up to 49% were also reported (1). Additionally, although plasma TFV concentrations are 20%–25% higher during the first 6 weeks postpartum than in the 3rd trimester, they remain lower than non-pregnant concentrations (4, 5). Lower TFV exposure during pregnancy is of particular concern, as meta-analyses, pooled study analyses, and pharmacometric modeling studies indicate that non-pregnant women already require higher drug concentrations required to achieve high levels of HIV protection in women compared to men (17–21). While plasma and PBMC concentrations of parent drugs (TFV, FTC) and active anabolites (TFV-DP, FTC-TP), respectively, are the same in men and women, drug deposition and TFV-DP concentrations are lower in cervicovaginal tissue as compared to colorectal tissue, which may contribute to the differences in TDF/FTC efficacy between men who have sex with men (MSM) vs. women (22–28).

We hypothesized that without doubling the TDF/FTC dose in pregnancy, substantial losses in HIV protection of 20%–40% would be expected due to moving down the concentration-response curve (17, 18, 29). The objective of the current analysis is to evaluate the effect of pregnancy on the pharmacokinetics (PK) of TDF and FTC in a population pharmacokinetics (popPK) modeling framework using a nonlinear mixed effects approach and to perform a clinical trial simulation to evaluate the appropriateness of a pregnancy-adjusted double TDF/FTC dose. Since the majority of TDF is rapidly converted to TFV after oral absorption, TFV is the primary circulating form of the drug in the plasma (30); thus the modeling and simulation were based on TFV plasma concentrations.

## Materials and methods

### Study design and study data

This analysis utilized popPK models of TFV and FTC and clinical trial simulation to compare the adequacy of standard TDF/FTC dosing to a pregnancy-adjusted, double TDF/FTC dose to maintain target plasma concentrations associated with HIV protection in the 1st, 2nd and 3rd trimesters of pregnancy. The pregnancy-adjusted double-dose TDF/FTC regimen was selected based on the demonstrated pregnancy-related concentration decreases in both TFV and FTC reported in the PK literature (1–9, 11–13, 15, 16).

We included data from two studies in the popPK modeling: the Partners Demonstration Project and data from the TDF/FTC arm of a phase I, prospective, open-label study conducted in Baltimore, Maryland by Coleman and colleagues (31, 32). The Partners Demonstration Project was a multi-site, randomized, double-blind, placebo-controlled clinical trial conducted in Kenya and Uganda, which included PK data from 116 female participants, including 33 pregnant and postpartum participants who became pregnant while taking TDF/FTC and elected to continue on TDF/FTC. TDF/FTC was provided in a MEMS® container, which records a time-and-date stamp for each container opening as a proxy for medication ingestion. The Coleman study included intensively sampled, steady-state PK data from 12 non-pregnant, pre-menopausal, HIV negative, cisgender women taking TDF/FTC under directly observed therapy (DOT). We chose the Partners Demonstration Project as it sampled the largest published cohort of pregnant and postpartum individuals on TDF/FTC PrEP. We included the Coleman, et al., PK study to supplement the Partners Demonstration Project PK data with intensive PK data under DOT. For both studies, plasma TFV and FTC concentrations were measured using a previously described, validated liquid chromatographic-tandem mass spectrometric (LC-MS/MS) assay (27). Lower limits of quantification (LLOQ) for plasma TFV and FTC were 0.31 ng/ml. All plasma drug concentrations were measured by the Clinical Pharmacology Analytical Laboratory at the Johns Hopkins University School of Medicine.

### Dataset preparation

We prepared the datasets for modeling by integrating MEMS data on adherence and TFV/FTC concentration data from the Partners Demonstration Project and dosing records from the Coleman et al. study. We used “M3” method articulated by Beal to handle drug concentrations below the limit of quantification (BLQ) in the Partner Demonstration Project. The M3 method accounts for measurements BLQ explicitly without censoring them. Thus, these observations are included in the PK model analysis using an appropriate statistical approach (33).

### Modeling and simulation

We conducted the population analysis using NONMEM (version 7.3. ICON Development Solution, USA) with the gfortran compiler interfaced with Perl-speaks-NONMEM (PsN). Dataset preparation and diagnostic plot plotting were carried out using R (4.1.1). The clinical simulation was carried out using mrgsolve package (1.0.8) in R.

### Model development

We developed the base model for TFV and FTC using the data from the Coleman et al. study. Based on the published models, we tested one-compartment and two-compartment models with first-order absorption and with or without lag time. After the development of the base model, we simultaneously used data from both the Coleman et al. study and the Partners Demonstration Project study for parameter estimation. For TFV, the exponential between subject variability was supported on first order absorption rate constant (Ka), apparent clearance (CL/F), apparent central (Vc/F) and peripheral volumes (Vp/F), and apparent inter-compartmental clearance (Q/F); For FTC, the exponential between subject variability was supported on Ka, CL/F, Vp/F, and Q/F:

$$P=\mathrm{TVP}\cdot \exp(\eta_{p})\;\;\;\;\eta_{p}∼N(0,\omega_{P}^{2})$$

Where the *P* represents the individual value of the parameter P, the TVP represent the typical value of the parameter P, the $\eta_{p}$ denotes the inter-individual variability (IIV) which is assumed to have a normal distribution with mean equals to 0 and variance equals to $\omega_{P}^{2}$.

For both TFV and FTC, we used a proportional residual model for the Coleman et al. study and a combined residual model for the Partners Demonstration Project to account for the heterogeneity of two clinical trials:

$$\begin{array}{c} C_{ij}=\hat{C_{ij}}\cdot (1+\varepsilon_{1ij}\cdot (2-\mathrm{STUDY})+\varepsilon_{2ij}\cdot (\mathrm{STUDY}-1))\\ \varepsilon_{3ij}\cdot (\mathrm{STUDY}-1)\\ \varepsilon_{1ij}∼N(0,\sigma_{1}^{\;2}),\varepsilon_{2ij}∼N(0,\sigma_{2}^{\;2}),\;\;\mathrm{and}\;\;\varepsilon_{3ij}∼N(0,\sigma_{3}^{\;2}) \end{array}$$

Where the $C_{\mathrm{ij}}$ represents the observed concentration of subject i at time j, the $\hat{C_{ij}}$ represents the predicted concentration, STUDY represents the study number (i.e., 1—Coleman et al. study, 2—Partners Demonstration Project). $\varepsilon_{1ij}$ and $\varepsilon_{2ij}$ represent the proportional error of data from the Coleman, et al. and the Partners Demonstration Project studies. $\varepsilon_{3ij}$ represents the additive error of data from the Partner Demonstration Project.

### Covariate evaluation

We tested potential covariates for TFV and FTC parameters, independently, using study number (i.e., 1 or 2 as above), baseline creatinine clearance, and pregnancy status. We treated pregnancy status as a categorical variable using 4 categories (0—non-pregnant, 1—1st trimester, 2—2nd trimester, and 3—3rd trimester). We evaluated different grouping methods on pregnancy data to test if the influence of each trimester could be identified separately. The pregnancy data were grouped as 1st trimester vs. 2nd trimester vs. 3rd trimester, 1st trimester and 2nd trimester vs. 3rd trimester, 1st trimester vs. 2nd trimester and 3rd trimester. Aggregation of all trimesters as a single factor was also tested. To assess covariate relationships, we first visualized the empirical Bayes estimates versus the potential covariates, and then employed stepwise selection method. For the forward selection, a decrease of the OFV more than 3.84 was considered significant for one degree of freedom (*p* < 0.05). For the backward elimination, an increase of OFV more than 6.63 was considered significant for one degree of freedom (*p* < 0.01).

### Model evaluation

We evaluated the performance of the final model by the diagnostic plots. This included evaluating the conditional weighted residuals and review of visual predictive checks. Concentrations associated with extreme deviations from the model prediction were assessed individually for physiologic plausibility. If an appropriate explanation of the outlier was not identified, the outlier was removed. A prediction corrected visual predictive check (pcVPC) of the final model showed the 5th, 50th, and 95th predicted percentiles from 1,000 simulated datasets with 128 individuals (12 from the Coleman et al. study and 116 from the Partners Demonstration Project), and generated the observed concentrations of TFV and FTC. The simulated concentrations that were BLQ were truncated to the LLOQ (0.31 ng/ml). We stratified the VPC by study.

### Clinical trial simulation

Based on the selected final population pharmacokinetic model, we conducted a clinical trial simulation to evaluate trough concentrations (C_trough_) of TFV and FTC during pregnancy. We simulated PK profiles of 1,000 cisgender female participants taking standard daily oral 300 mg TDF/200 mg FTC prior to pregnancy. Upon becoming pregnant, simulated participants were split into two arms: arm 1 (*n* = 500) continuing the standard dose regimen and arm 2 (*n* = 500) receiving a pregnancy-adjusted, double-dose of both TFV and FTC. We assumed an increase in renal clearance due to pregnancy beginning in the 1st trimester. Simulated trough plasma concentrations of TFV and FTC were compared with the lower bound of expected trough concentration benchmarks, estimated to be the protective thresholds associated with daily dosing estimated from HPTN 066, 35.5 ng/ml for TFV and 49.1 ng/ml for FTC (28).

## Results

The final dataset included data from 128 women (12 from the Coleman, et al., study and 116 from the Partners Demonstration Project; see Table 1). Data included 33 pregnant women, of whom, 29, 24, and 23 women contributed data from their 1st, 2nd, and 3rd trimesters, respectively. For TFV, there are 39 (6 BLQ) samples in the 1st trimester, 59 (14 BLQ) samples in the 2nd trimester, and 62 (20 BLQ) samples in the 3rd trimester. For FTC, there are 37 (9 BLQ) samples in the 1st trimester, 55 (15 BLQ) samples in the 2nd trimester, and 55 (22 BLQ) samples in the 3rd trimester. Total concentrations available for modeling included 487 TFV and 465 FTC measurements. Upon visual exploration of the final model, outliers were noted in the pcVPC. Further examination revealed four TFV measurements (0.82% of the total measures) and twelve FTC measurements (2.6% of total measures) that were physiologically implausible. These were removed from the dataset and the population model re-run. Minor differences were noted for TFV in the CL (51.5 vs. 52.4 L/h) and Vp/F (1,160 L vs. 1,120 L). A larger change was observed in the Vc/F (359 vs. 252 L).

**Table 1:** Participant demographics from the partners demonstration project and the coleman et al. study.

Parameter	Partners demonstration		Coleman et al.
	Non-pregnant	Pregnant	Non-pregnant
Number of participants	97 (83 with plasma samples)	37 (33 with plasma samples)	12
Race^a^	–	–	9B, 2W, 1A
Ethnicity^a^	–	–	1H
	mean (SD)		median (IQR)
Age	30.6 (7.4)	25.1 (4.8)	34 (28–37)
Weight (kg)	–	–	90 (78–101)
BMI	24.5 (4.3)	24.6 (4.7)	–
CrCl (ml/min)	101.8 (18.4)	111.7 (27.5)	139 (115–172)
iGFR (ml/min/1.73 m^2^)			102 (88–114)

^a^ Black (B), White (W), Asian (A), Hispanic (H).

In the FTC population model, CL did not change; however, the central volume of distribution changed from 90.7 to 66.9 L and the peripheral volume of distribution changed from 195 to 166 L.

### TFV model

**Figure 1:**
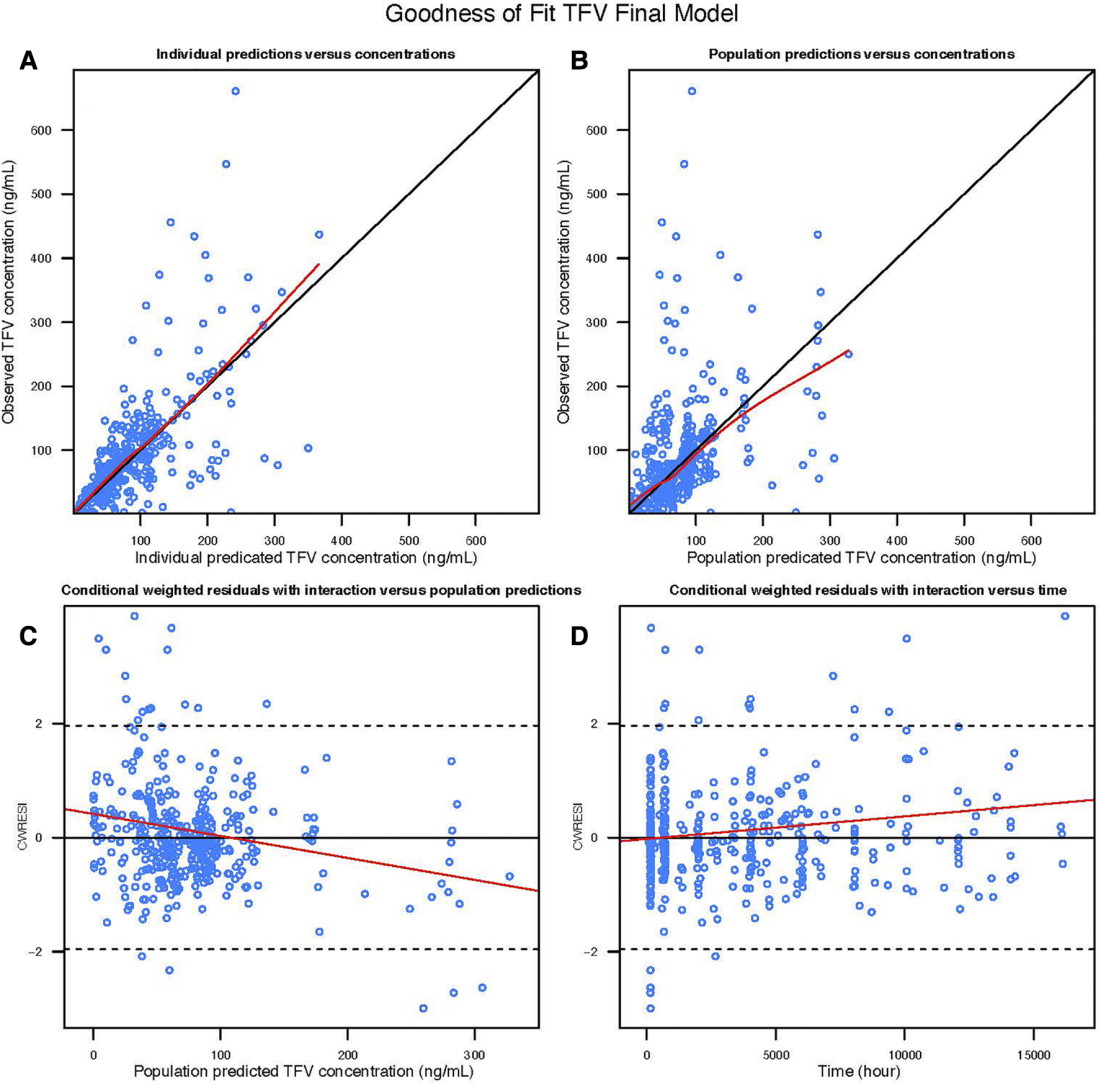
Goodness of fit plot of the TFV final model: (**A**) observed TFV concentration vs. Individual predicted TFV concentration; (**B**) observed TFV concentration vs. population predicted TFV concentration; (**C**) conditional weighted residuals with interaction vs. population predicted TFV concentration; (**D**) conditional weighted residuals with interaction vs. time.

**Figure 2:**
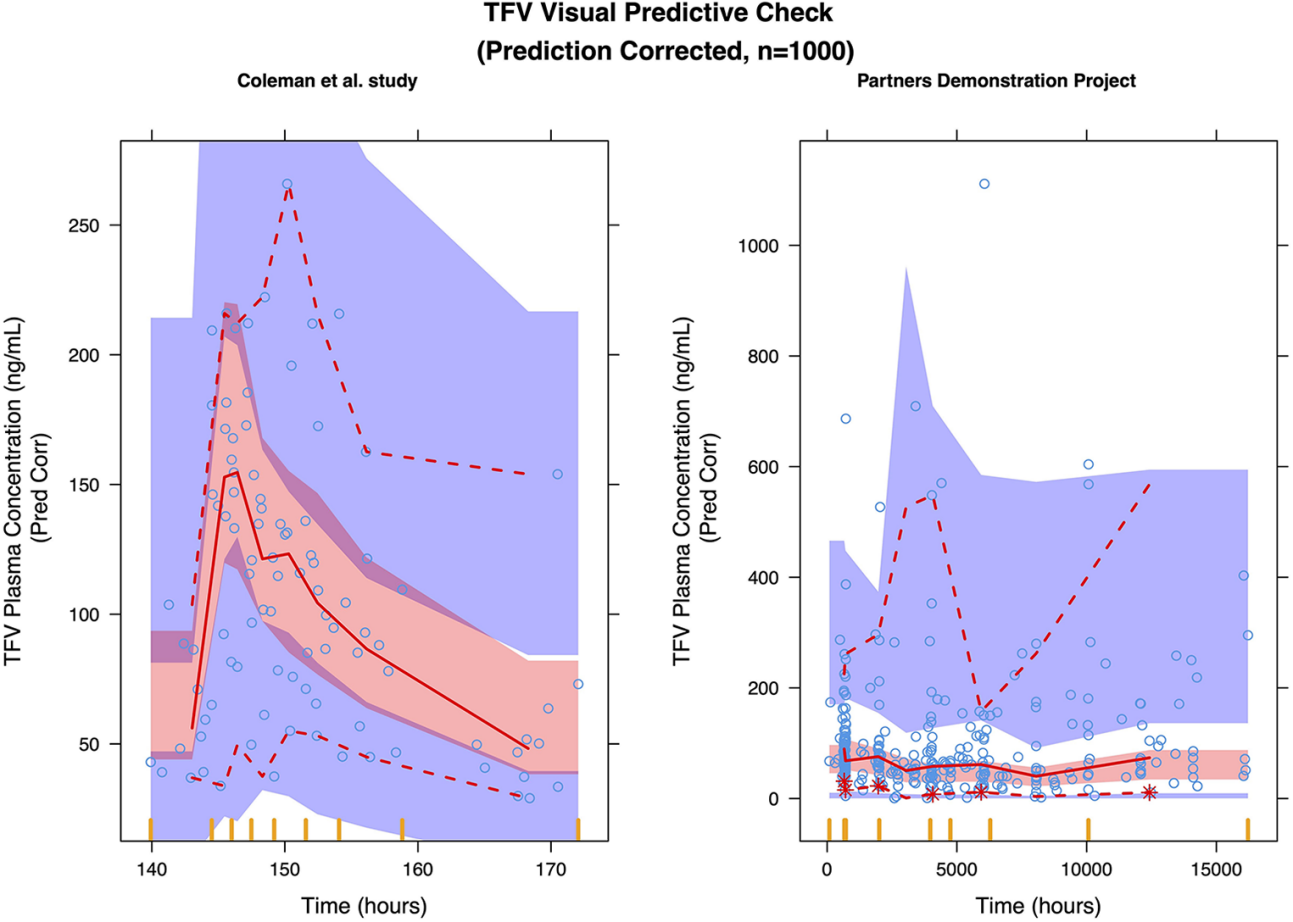
Prediction corrected visual predictive check (pcVPC) of the TFV final model: (left) coleman, et al. study; (right) partners demonstration project.

A two-compartment model with first order absorption adequately described the pharmacokinetics of TFV in this population (Table 2); the diagnostic plots and VPCs indicated good agreement between observed and predicted values (Figures 1, 2). Our final model overestimated the TFV trough concentration in the Coleman et al. study, as seen in Figure 2. Inclusion of trimester as a covariate in the apparent clearance significantly reduced the objective function value (OFV) by 48.809. The typical value of the apparent clearance of TFV increased by 1.214, 1.339, and 1.639-fold in the 1st, 2nd, and 3rd trimester, respectively, compared to the non-pregnant baseline values; these data are comparable to the previously reported clearance increment during each trimester. The proportional error of the Partners Demonstration Project (71.2%) was higher than the Coleman et al. study (21.2%).

**Table 2:** Final estimates of TFV pharmacokinetic parameters, between subject variability, and residual variability^a^.

Parameter	Estimate	RSE%
CL/F (L/h)	52.4	7
V2/F (L)	252	57
Q/F (L/h)	295	18
V3/F (L/h)	1120	18
KA (/h)	2.56	91
CL/F increment during 1st trimester (%)	21.4%	55
CL/F increment during 2nd trimester (%)	33.9%	34
CL/F increment during 3rd trimester (%)	63.9%	29
BSV on CL/F	35.9%	13
BSV on V2/F	41.4%	145
BSV on Q/F	67.6%	29
BSV on V3/F	56.7%	28
BSV on KA	56.4%	87
σ1(prop; Coleman study)	21.2%	6
σ2(prop; partner demonstration project)	71.2%	5
σ2(add; partner demonstration project) (ng/ml)	0.109	253

^a^ CL/F, apparent clearance; V2/F, apparent volume of distribution of the central compartment; Q/F, apparent intercompartmental clearance; V3/F, apparent volume of distribution of the peripheral compartment; KA, absorption rate constant; BSV, between-subject variability; σ1, proportional residual error; σ2, additive residual error; RSE, relative standard error.

### FTC model

**Figure 3:**
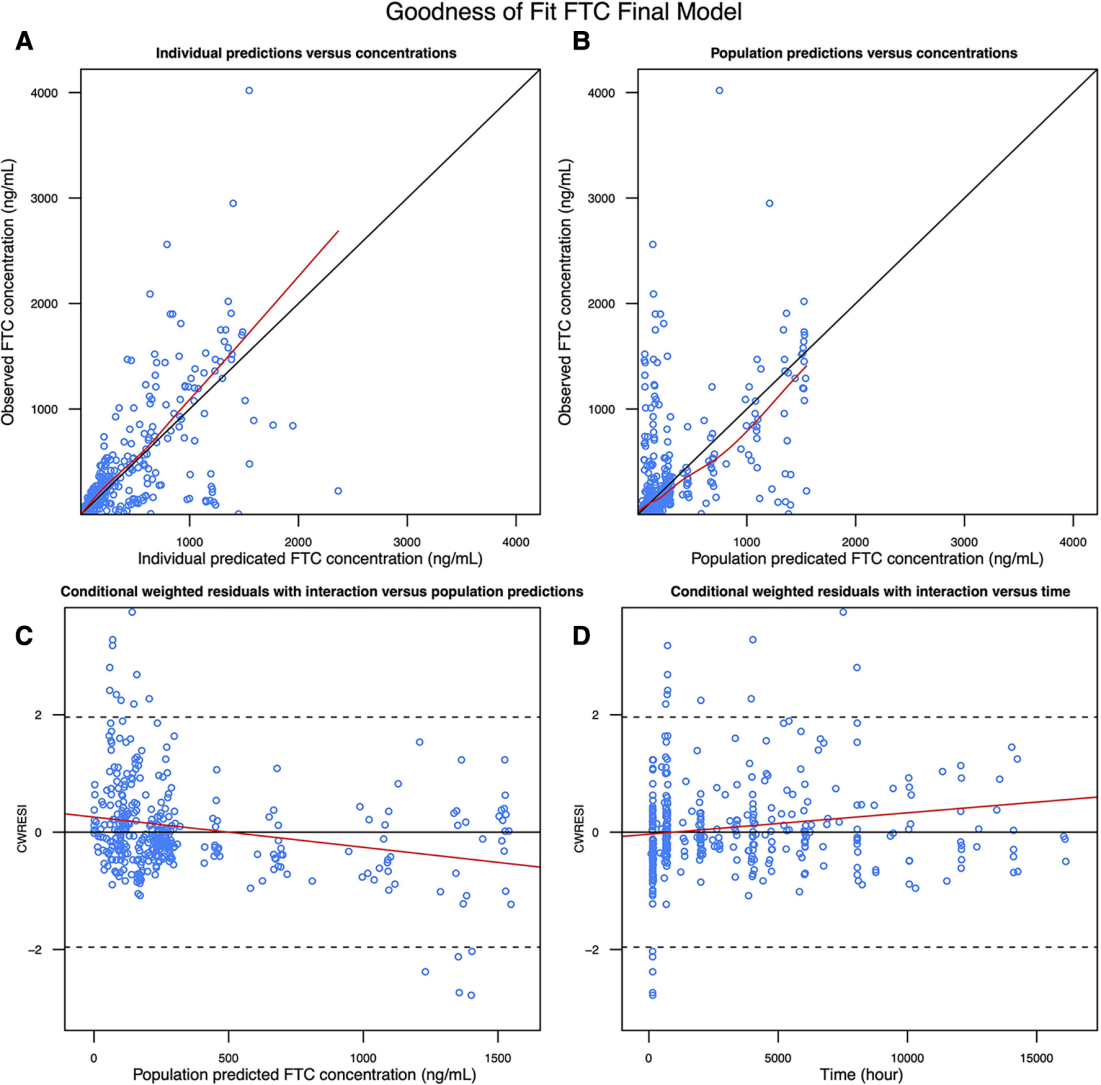
Goodness of fit plot of the FTC final model: (**A**) observed FTC concentration vs. Individual predicted FTC concentration; (**B**) observed FTC concentration vs. population predicted FTC concentration; (**C**) conditional weighted residuals with interaction vs. population predicted FTC concentration; (**D**) conditional weighted residuals with interaction vs. time.

**Figure 4:**
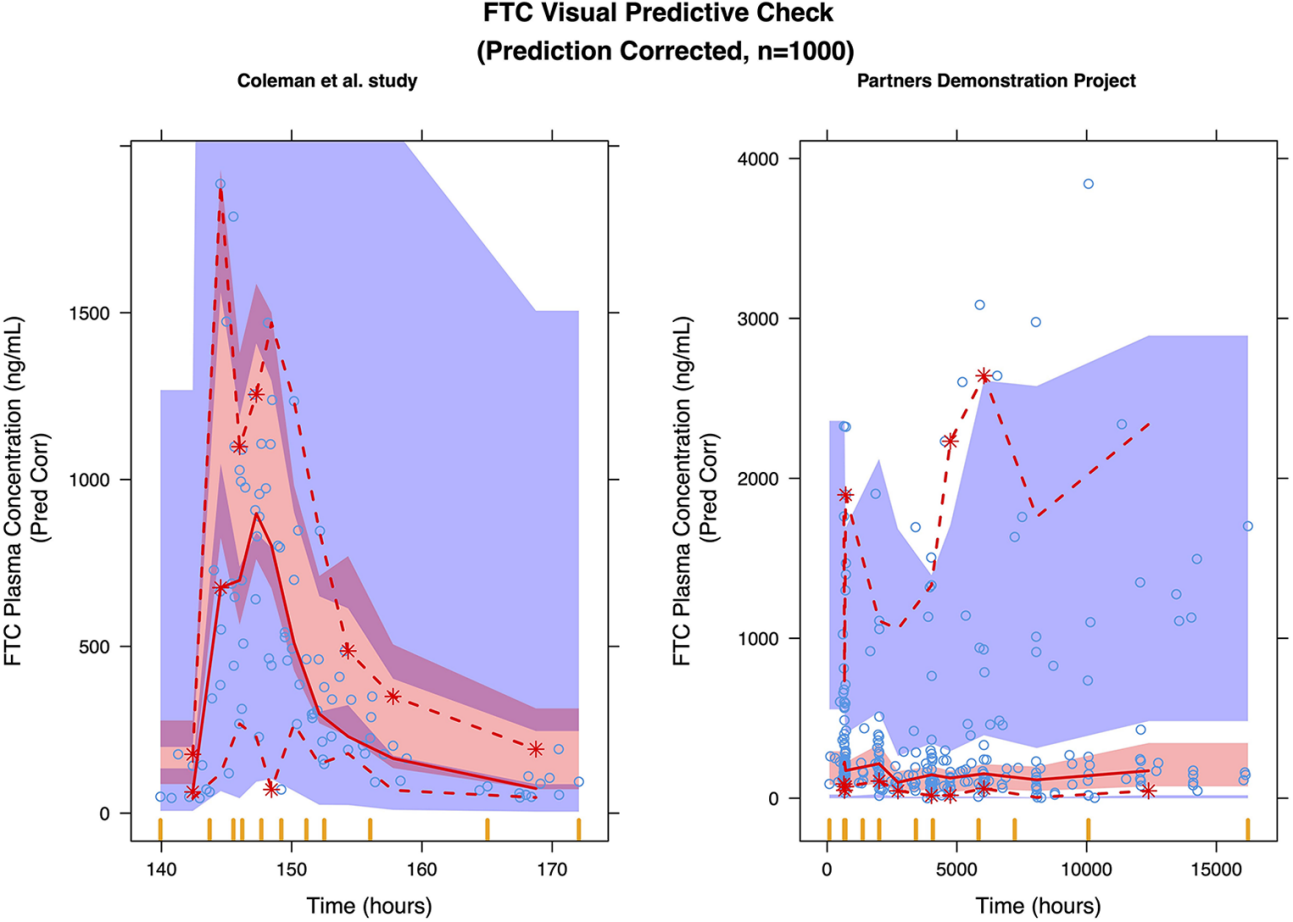
Prediction corrected visual predictive check (pcVPC) of the FTC final model: (left) coleman et al. study; (right) partners demonstration project.

We selected a two-compartment model with first-order absorption as the final structural model (Table 3). Since the simulated changes in FTC clearance (compared to pre-pregnancy) for each trimester were commensurate with the change when from pre-pregnancy to pregnancy (all trimesters combined), we used combined data from all trimesters in the final model. Pregnancy increased the apparent clearance by 63.1% compared to the non-pregnant baseline value, reducing the OFV by 27.685. As with TFV, we found a high proportional error of the Partners Demonstration Project data (85.4%). The diagnostic plot (Figure 3) showed some bias. The VPC (Figure 4) indicated the satisfactory performance of the final model. Our final model overestimated FTC plasma concentrations compared to those found in the Coleman et al. study.

**Table 3:** Final estimates of FTC pharmacokinetic parameters, between subject variability, and residual variability^a^.

Parameter	Estimate	RSE%
CL/F (L/h)	16.7	9
V2/F (L)	58.8	62
Q/F (L/h)	13.8	22
V3/F (L/h)	190	18
KA (/h)	0.616	56
CL/F increment during pregnancy (%)	63.1%	23
BSV on CL/F	50.6%	9
BSV on Q/F	62.5%	43
BSV on V3/F	41.6%	60
BSV on KA	20.5%	26
σ1(prop; Coleman study)	29.3%	8
σ2(prop; partner demonstration project)	85.4%	6
σ2(add; partner demonstration project) (ng/ml)	12.5	31

^a^ CL/F, apparent clearance; V2/F, apparent volume of distribution of the central compartment; Q/F, apparent intercompartmental clearance; V3/F, apparent volume of distribution of the peripheral compartment; KA, absorption rate constant; BSV, between-subject variability; σ1, proportional residual error; σ2, additive residual error; RSE, relative standard error.

### Clinical trial simulation

**Figure 5:**
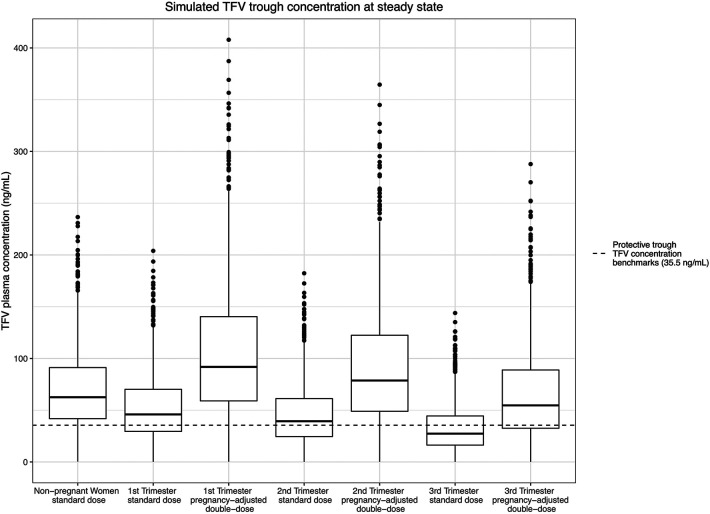
Simulated TFV trough concentration at steady state and the estimated protective trough concentration of TFV.

In the non-pregnant population, the simulated median steady-state trough plasma concentration was 62.5 ng/ml for TFV and 158 ng/ml for FTC. Our simulation indicated that 13.9% and 16.4% of the participants on a standard “pre-pregnancy” regimen would have steady-state trough plasma TFV and FTC concentrations below the estimated protective threshold, respectively. In the standard TDF/FTC dosing arm (arm 1), the simulated median steady-state plasma TFV trough concentration dropped to 45.9 ng/ml, 39.3 ng/ml, and 27.3 ng/ml in the 1st, 2nd, and 3rd trimesters, respectively. According to our simulations, steady-state median TFV plasma concentrations decrease by 26.5–56.3% throughout pregnancy from a pre-pregnant baseline. Accordingly, we found that 34.0%, 43.8%, and 65.1% of steady-state plasma trough concentrations dropped below the estimated protective TFV trough concentration (35.5 ng/ml) due to the progressively increased clearance in the three trimesters. By comparison, in the simulated arm 2 pregnancy-adjusted double-dose group, the simulated median steady-state plasma trough concentration were 91.8 ng/ml, 78.7 ng/ml, and 54.6 ng/ml in the 1st, 2nd, and 3rd trimesters. Only 10.7%, 14.4%, and 27.8% of participants in the pregnancy-adjusted double-dose arm had steady-state trough plasma concentrations less than 35.5 ng/ml (Figure 5).

**Figure 6:**
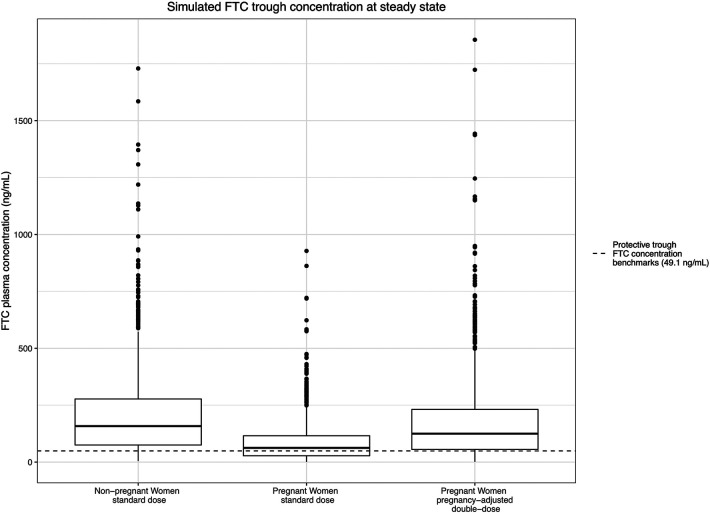
Simulated FTC trough concentration at steady state and the estimated protective trough concentration of FTC.

For FTC, since all trimesters were combined in the final model, the simulated steady-state trough concentration estimates trough concentrations throughout pregnancy. In the arm 1 typical dosing group, the median simulated steady-state trough plasma concentration during the pregnant period was 62.4 ng/ml During pregnancy, 42.1% of the steady-state trough concentrations dropped below the estimated protective trough concentrations for FTC (49.1 ng/ml). In the pregnancy-adjusted double-dose arm, the median simulated steady-state trough plasma concentration was 125 ng/ml; 22.4% of participants had trough concentrations less than 49.1 ng/ml, similar to the non-pregnancy group (Figure 6).

## Discussion

We analyzed sparsely sampled PK data from the Partners Demonstration Project and intensively sampled PK data from the Coleman et al. study for both plasma TFV and FTC using a nonlinear modeling framework. Removal of outlier values had a modest impact on the popPK parameter estimates, but resulted in significantly improved model performance measures (pcVPC). The central volume of distribution estimate after removal of the outliers is consistent with that reported in the literature (15, 34).

We observed a progressive increase in clearance for TFV throughout pregnancy, with a nearly two-fold increase in clearance in the 3rd trimester compared to the non-pregnant baseline, and an associated progressive decrease in trough plasma levels. For FTC, we observed a smaller increase in clearance in pregnancy, and an associated decrease in trough concentrations; these changes were consistent throughout pregnancy. Clinical trial simulation of standard vs. pregnancy-adjusted double-dose TDF/FTC regimens revealed that, compared to non-pregnant women, a clinically significant proportion of pregnant individuals on the standard dose would have exposures below the estimated protective thresholds for both TFV (35.5 ng/ml) and FTC (49.1 ng/ml) in part or all, respectively, of pregnancy. In contrast, the pregnancy-adjusted dosing regimen significantly reduced the proportion of pregnant individuals falling below the estimated protective threshold from 34%, 43.8%, and 65.1%, to 10.7%, 14.4%, and 27.8% for TFV during the 1st, 2nd, and 3rd trimesters and from 42.1% to 22.4% for FTC during pregnancy. For context, the simulated 1st trimester steady-state plasma TFV trough concentrations with doubled TDF/FTC dosing—10.7% below and 89.3% above the 35.5 ng/ml daily dosing benchmark—is consistent with the 90% sensitivity threshold used in HPTN 066 to select the 35.5 ng/ml benchmark. Even so, the doubled TDF/FTC daily dose did not fully correct plasma TFV in the 2nd and 3rd trimester or FTC during pregnancy to pre-pregnant levels.

Consistent with our findings, physiological changes in renal blood flow are known to be progressive in pregnancy and are associated with progressive increases in clearance and decreases in exposure for renally excreted drugs, such as TFV and FTC. Although not identified in our final model, the increased volume of distribution during pregnancy may also contribute to lower plasma concentrations of TFV and FTC. The 26.5–56.3% reduction we estimated in simulated TFV trough plasma concentrations throughout the pregnancy is consistent with the 45%–58% reduction in TFV concentration reported by Pyra et al. in the averaged TFV concentrations in the Partners Demonstration Project (1). A popPK analysis by Benaboud et al. found a 39% increased clearance during pregnancy in women with HIV on TDF/FTC-containing regimens (2). A whole body physiologically based pharmacokinetic (PBPK) model by De Sousa Mendes et al. in pregnancy predicted a 40% increase in TFV apparent clearance at approximately 33 weeks gestational age (35). A popPK model in women with HIV during pregnancy developed by Hirt et al. showed up to a 50% increase in the apparent clearance of FTC compared with the non-pregnant population (11), similar to our final estimates. A simplified pregnant-PBPK model developed by Xia et al. predicted a 1.39-fold change in the renal clearance of FTC in late pregnancy due to increased in renal secretion and filtration (36). The whole body PBPK model mentioned above predicted a 1.29-fold clearance change, which is slightly lower than our estimates (35). Liu et al. predicted the PK profiles of FTC at different stages of pregnancy using a maternal-fetal PBPK model. They predicted an up to 27.7% decrease in median FTC AUC at 26 weeks of gestation (37).

Despite the availability of newer PrEP modalities, TDF/FTC remains the main stay of HIV prevention in pregnancy. Although there are safety and PK data for HIV treatment in cisgender women, including during pregnancy, tenofovir alafenamide (TAF)/FTC is not yet recommended in cisgender women for PrEP given the lack of efficacy data. There are only limited safety and PK data for long-acting Cabotegravir in pregnancy (38, 39) and although there are reassuring safety data on the use of the Dapivirine ring in pregnancy (40, 41), its approval is limited globally. Decreased protective efficacy of TDF/FTC PrEP during pregnancy due to lower TFV and FTC exposures, as indicated in the clinical trial simulation, is cause for considerable concern, especially as the baseline HIV incidence among pregnant and postpartum women is two to four times that of non-pregnant women (42, 43). Modeled infectivity from the Partners in Prevention HSV/HIV Transmission Study and the Partners PrEP study demonstrated that the probability of HIV acquisition per condomless sex act increases starting in early pregnancy and peaks postpartum [adjusted RR 3.97 (1.50, 10.51) *p* < 0.001]) (43). Data from several observational studies corroborate that model’s findings of increased male-to-female transmission in pregnancy (42–48). This increased incidence is attributed to both behavioral and biological changes (including immunological, vaginal microbiome, and vaginal epithelial integrity) during pregnancy and delivery (42, 46, 49–51). Prevention of HIV is especially critical in pregnant individuals secondary to the additional and increased risk of perinatal transmission. The risk of perinatal transmission is 9–15-fold higher in women diagnosed with HIV during (vs. prior to) pregnancy (22 vs. 1.8%) (52, 53). Increased HIV acquisition attributable to decreased protection of TDF/FTC PrEP against HIV during pregnancy has not been reported, but it is unclear if this is due to the adequacy of TDF/FTC PrEP protection in pregnancy vs. underutilization of PrEP in pregnancy and a dearth of large-scale research on PrEP in pregnancy. Limited clinical trials and epidemiologic research have focused on oral PrEP in pregnancy, but none in sufficient size to evaluate increased incidence due to TDF/FTC PrEP failure.

Limitations of the current analysis include the availability in pregnancy of only sparse PK data and only plasma drug concentrations, rather than active intracellular phosphorylated analytes. Additionally, we did not include body weight and renal clearance as covariates or intracellular metabolite concentrations in our model. Neither study controlled for diet nor timing of dose related to meals, which could introduce additional variability (54, 55). Regarding the differences in CrCl between populations, kidney estimation equations were primarily derived in non-Black populations, and the equations used in the United States at the time the original data were collected (e.g., Coleman et al.) are not always applicable to African populations (e.g., Partners Demonstration Project). Previously published models found body weight (10) and creatinine clearance (10, 34, 56–58) to be significant covariates for TFV and FTC clearance. Even without inclusion of these covariates, the model still captures the global effect of trimester on clearance for TFV and underscores the need for a pooled analysis of all clinical trial data in pregnancy to better understand the dose optimization needs and for prospective PK research on dosing in pregnancy. For FTC, we were unable to identify the different changes in its clearance over different trimesters. An additional limitation was the need for a separate residual error model for Partners Demonstration Project; of particular concern was the large proportional error potentially attributable to differences in PK sampling and ascertainment of the dosing history. During our clinical trial simulation, we discovered that up to 65.1% of the TFV trough concentration and 45.2% of the FTC trough concentration in pregnant population may fall below the protective threshold. However, we also observed that our final model tended to overestimate the TFV and FTC concentration in the non-pregnant population. As a result, the proportion of pregnant individuals with trough concentrations below the protective threshold (based on empiric observations) may have been underestimated. An additional limitation is that although Partners Demonstration Project utilized MEMS to measure adherence, doses were not observed and activation of the MEMS without taking a dose or taking a double dose (“catch up dosing”) prior to a study visit could bias CL/F. Lastly, as noted above, our sample size and that of published studies are insufficient to assess any impact of pregnancy on TDF/FTC PrEP efficacy.

Our popPK model and clinical trial simulation found that steady-state TFV and FTC trough plasma concentrations decreased during pregnancy, which puts pregnant individuals receiving standard TDF/FTC dosing at significantly greater risk of falling below the protective thresholds for both TFV and FTC compared to participants taking the pregnancy-adjusted double dose. This simulation provides the quantitative basis for the design of prospective TDF/FTC studies during pregnancy to evaluate the safety and appropriateness of pregnancy-adjusted dosing.

## Data Availability

The data analyzed in this study is subject to the following licenses/restrictions: Available upon request. Requests to access these datasets should be directed to chendrix@jhmi.edu.

## References

[1] Pyra M Anderson PL Hendrix CW Heffron R Mugwanya K Haberer JE Tenofovir and tenofovir-diphosphate concentrations during pregnancy among HIV-uninfected women using oral preexposure prophylaxis. AIDS. (2018) 32(13):1891–8. 10.1097/QAD.0000000000001922 29894385 PMC6061961

[2] Benaboud S Hirt D Launay O Pannier E Firtion G Rey E Pregnancy-related effects on tenofovir pharmacokinetics: a population study with 186 women. Antimicrob Agents Chemother. (2012) 56(2):857–62. 10.1128/AAC.05244-11 22123690 PMC3264275

[3] Flynn PM Mirochnick M Shapiro DE Bardeguez A Rodman J Robbins B Pharmacokinetics and safety of single-dose tenofovir disoproxil fumarate and emtricitabine in HIV-1-infected pregnant women and their infants. Antimicrob Agents Chemother. (2011) 55(12):5914–22. 10.1128/AAC.00544-11 21896911 PMC3232794

[4] Colbers APH Hawkins DA Gingelmaier A Kabeya K Rockstroh JK Wyen C The pharmacokinetics, safety and efficacy of tenofovir and emtricitabine in HIV-1-infected pregnant women. AIDS. (2013) 27(5):739–48. 10.1097/QAD.0b013e32835c208b 23169329

[5] Best B Burchett S Li H Stek A Hu C Wang J Pharmacokinetics of tenofovir during pregnancy and postpartum. HIV Med. (2015) 16(8):502–11. 10.1111/hiv.12252 25959631 PMC4862736

[6] Hirt D Ekouévi DK Pruvost A Urien S Arrivé E Blanche S Plasma and intracellular tenofovir pharmacokinetics in the neonate (ANRS 12109 trial, step 2). Antimicrob Agents Chemother. (2011) 55(6):2961–7. 10.1128/AAC.01377-10 21464249 PMC3101430

[7] Zong J Chittick GE Wang LH Hui J Begley JA Blum MR. Pharmacokinetic evaluation of emtricitabine in combination with other nucleoside antivirals in healthy volunteers. J Clin Pharmacol. (2007) 47(7):877–89. 10.1177/0091270007300808 17526857

[8] Blum MR Chittick GE Begley JA Zong J. Steady-State pharmacokinetics of emtricitabine and tenofovir disoproxil fumarate administered alone and in combination in healthy volunteers. J Clin Pharmacol. (2007) 47(6):751–9. 10.1177/0091270007300951 17519400

[9] Ramanathan S Shen G Cheng A Kearney BP. Pharmacokinetics of emtricitabine, tenofovir, and GS-9137 following coadministration of emtricitabine/tenofovir disoproxil fumarate and ritonavir-boosted GS-9137. J Acquir Immune Defic Syndr. (2007) 45(3):274–9. 10.1097/QAI.0b013e318050d88c 17414929

[10] Jullien V TréLuyer J-M Rey E Jaffray P Krivine A Moachon L Population pharmacokinetics of tenofovir in human immunodeficiency virus-infected patients taking highly active antiretroviral therapy. Antimicrob Agents Chemother. (2005) 49(8):3361–6. 10.1128/AAC.49.8.3361-3366.2005 16048948 PMC1196246

[11] Hirt DB Urien S Rey E Arrivé E EkouéVi DK Coffié P Population pharmacokinetics of emtricitabine in human immunodeficiency virus type 1-infected pregnant women and their neonates. Antimicrob Agents Chemother. (2009) 53(3):1067–73. 10.1128/AAC.00860-08 19104016 PMC2650537

[12] Duwal S Schütte C Von Kleist M. Pharmacokinetics and pharmacodynamics of the reverse transcriptase inhibitor tenofovir and prophylactic efficacy against HIV-1 infection. PLoS One. (2012) 7(7):e40382. 10.1371/journal.pone.0040382 22808148 PMC3394807

[13] Chaturvedula A Fossler MJ Hendrix CW. Estimation of tenofovir’s population pharmacokinetic parameters without reliable dosing histories and application to tracing dosing history using simulation strategies. J Clin Pharmacol. (2014) 54(2):150–60. 10.1002/jcph.221 24203458 PMC5001555

[14] Dumond JB Yeh RF Patterson KB Corbett AH Jung BH Rezk NL Antiretroviral drug exposure in the female genital tract: implications for oral pre- and post-exposure prophylaxis. AIDS. (2007) 21(14):1899–907. 10.1097/QAD.0b013e328270385a 17721097 PMC2862268

[15] Baheti G Kiser JJ Havens PL Fletcher CV. Plasma and intracellular population pharmacokinetic analysis of tenofovir in HIV-1-infected patients. Antimicrob Agents Chemother. (2011) 55(11):5294–9. 10.1128/AAC.05317-11 21896913 PMC3194996

[16] Mirochnick M Taha T Kreitchmann R Nielsen-Saines K Kumwenda N Joao E Pharmacokinetics and safety of tenofovir in HIV-infected women during labor and their infants during the first week of life. J Acquir Immune Defic Syndr. (2014) 65(1):33–41. 10.1097/QAI.0b013e3182a921eb 23979002 PMC3912736

[17] Hendrix. Exploring concentration response in HIV pre-exposure prophylaxis to optimize clinical care and trial design. Cell. (2013) 155(3):515–8. 10.1016/j.cell.2013.09.030 24243011

[18] Cottrell ML Yang KH Prince HM Sykes C White N Malone S A translational pharmacology approach to predicting outcomes of preexposure prophylaxis against HIV in men and women using tenofovir disoproxil fumarate with or without emtricitabine. J Infect Dis. (2016) 214(1):55–64. 10.1093/infdis/jiw077 26917574 PMC4907409

[19] Hanscom B Janes HE Guarino PD Huang Y Brown ER Chen YQ Brief report: preventing HIV-1 infection in women using oral preexposure prophylaxis: a meta-analysis of current evidence. JAIDS J Acquir Immune Defic Syndr. (2016) 73(5):606–8. 10.1097/QAI.0000000000001160 27846073 PMC5175411

[20] Jayachandran P Garcia-Cremades M Vučićević K Bumpus NN Anton P Hendrix C A mechanistic in vivo / ex vivo pharmacokinetic-pharmacodynamic model of tenofovir for HIV prevention. CPT Pharmacometrics Syst Pharmacol. (2021) 10(3):179–87. 10.1002/psp4.12583 33547874 PMC7965838

[21] Garcia-Cremades M Vučićević K Hendrix CW Jayachandran P Jarlsberg L Grant R Characterizing HIV-preventive, plasma tenofovir concentrations-a pooled participant-level data analysis from human immunodeficiency virus preexposure prophylaxis clinical trials. Clin Infect Dis. (2022) 75(11):1873–82. 10.1093/cid/ciac313 35474481 PMC10139701

[22] Patterson KB Prince HA Kraft E Jenkins AJ Shaheen NJ Rooney JF Penetration of tenofovir and emtricitabine in mucosal tissues: implications for prevention of HIV-1 transmission. Sci Transl Med. (2011) 3(112):112re4–re4. 10.1126/scitranslmed.3003174 22158861 PMC3483088

[23] Louissaint NA Cao Y-J Skipper PL Liberman RG Tannenbaum SR Nimmagadda S Single dose pharmacokinetics of oral tenofovir in plasma, peripheral blood mononuclear cells, colonic tissue, and vaginal tissue. AIDS Res Hum Retrovir. (2013) 29(11):1443–50. 10.1089/aid.2013.0044 23600365 PMC3809387

[24] Seifert SM Chen X Meditz AL Castillo-Mancilla JR Gardner EM Predhomme JA Intracellular tenofovir and emtricitabine anabolites in genital, rectal, and blood compartments from first dose to steady state. AIDS Res Hum Retrovir. (2016) 32(10–11):981–91. 10.1089/aid.2016.0008 27526873 PMC5067852

[25] Thurman AR Schwartz JL Cottrell ML Brache V Chen BA Cochón L Safety and pharmacokinetics of a tenofovir alafenamide fumarate-emtricitabine based oral antiretroviral regimen for prevention of HIV acquisition in women: a randomized controlled trial. EClinicalMedicine. (2021) 36:100893. 10.1016/j.eclinm.2021.100893 34041459 PMC8144741

[26] Ouattara LA Thurman AR Jacot TA Cottrell M Sykes C Blake K Genital mucosal drug concentrations and anti-HIV activity in tenofovir-based PrEP products: intravaginal ring vs. oral administration. J Acquir Immune Defic Syndr. (2022) 89(1):87–97. 10.1097/QAI.0000000000002820 34878438 PMC8647693

[27] Hendrix CW Chen BA Guddera V Hoesley C Justman J Nakabiito C MTN-001: randomized pharmacokinetic cross-over study comparing tenofovir vaginal gel and oral tablets in vaginal tissue and other compartments. PLoS One. (2013) 8(1):e55013. 10.1371/journal.pone.0055013 23383037 PMC3559346

[28] Hendrix CW Andrade A Bumpus NN Kashuba AD Marzinke MA Moore A Dose frequency ranging pharmacokinetic study of tenofovir-emtricitabine after directly observed dosing in healthy volunteers to establish adherence benchmarks (HPTN 066). AIDS Res Hum Retrovir. (2016) 32(1):32–43. 10.1089/aid.2015.0182 26414912 PMC4692123

[29] Duwal S von Kleist M. Top-down and bottom-up modeling in system pharmacology to understand clinical efficacy: an example with NRTIs of HIV-1. Eur J Pharm Sci. (2016) 94:72–83. 10.1016/j.ejps.2016.01.016 26796142

[30] Wassner C Bradley N Lee Y. A review and clinical understanding of tenofovir: tenofovir disoproxil fumarate versus tenofovir alafenamide. J Int Assoc Provid AIDS Care. (2020) 19:232595822091923. 10.1177/2325958220919231 PMC7163232 32295453

[31] Nakku-Joloba E Pisarski EE Wyatt MA Muwonge TR Asiimwe S Celum CL Beyond HIV prevention: everyday life priorities and demand for PrEP among Ugandan HIV serodiscordant couples. J Int AIDS Soc. (2019) 22(1):e25225. 10.1002/jia2.25225 30657642 PMC6338102

[32] Coleman JS Diniz CP Fuchs EJ Marzinke MA Aung W Bakshi RP Interaction of depot medroxyprogesterone acetate and tenofovir disoproxil fumarate/emtricitabine on peripheral blood mononuclear cells and cervical tissue susceptibility to HIV infection and pharmacokinetics. J Acquir Immune Defic Syndr. (2023) 92(1):89–96. 10.1097/QAI.0000000000003113 36305827 PMC9742287

[33] Beal SL. Ways to fit a PK model with some data below the quantification limit. J Pharmacokinet Pharmacodyn. (2001) 28(5):481–504. 10.1023/A:1012299115260 11768292

[34] Tanaudommongkon A Chaturvedula A Hendrix CW Fuchs EJ Shieh E Bakshi RP Population pharmacokinetics of tenofovir, emtricitabine and intracellular metabolites in transgender women. Br J Clin Pharmacol. (2022) 88(8):3674–82. 10.1111/bcp.15310 35285974 PMC9296590

[35] Mendes M DS Hirt D Urien S Valade E Bouazza N Foissac F Physiologically-based pharmacokinetic modeling of renally excreted antiretroviral drugs in pregnant women. Br J Clin Pharmacol. (2015) 80(5):1031–41. 10.1111/bcp.12685 26011128 PMC4631176

[36] Xia B Heimbach T Gollen R Nanavati C He H. A simplified PBPK modeling approach for prediction of pharmacokinetics of four primarily renally excreted and CYP3A metabolized compounds during pregnancy. AAPS J. (2013) 15(4):1012–24. 10.1208/s12248-013-9505-3 23835676 PMC3787241

[37] Liu XI Momper JD Rakhmanina N Den Anker JN Green DJ Burckart GJ Physiologically based pharmacokinetic models to predict maternal pharmacokinetics and fetal exposure to emtricitabine and acyclovir. J Clin Pharmacol. (2020) 60(2):240–55. 10.1002/jcph.1515 31489678 PMC7316130

[38] Delany-Moretlwe JHS Guo X Hanscom B Hendrix CW Farrior J Berhanu RH Evaluation of CAB-LA safety and PK in pregnant women in the blinded phase of HPTN 084 [CROI abstract 700]. In special issue: abstracts from the 2022 conference on retroviruses and opportunistic infections. Top Antivir Med. (2022) 30(1s):382.

[39] Patel P Ford SL Baker M Meyer C Garside L D'Amico R Pregnancy outcomes and pharmacokinetics in pregnant women living with HIV exposed to long-acting cabotegravir and rilpivirine in clinical trials. HIV Med. (2023) 24(5):568–79. 10.1111/hiv.13439 36411596

[40] Makanani B Balkus JE Jiao Y Noguchi LM Palanee-Phillips T Mbilizi Y Pregnancy and infant outcomes among women using the dapivirine vaginal ring in early pregnancy. J Acquir Immune Defic Syndr. (2018) 79(5):566–72. 10.1097/QAI.0000000000001861 30383589 PMC6231990

[41] Bunge KE Bunge JB Mhlanga F Mayo A Fairlie L Nakabiito C Deliver: a safety study of a dapivirine vaginal ring and oral PrEP during pregnancy [CROI abstract 127]. In special issue: abstracts from the 2023 conference on retroviruses and opportunistic infections. Top Antivir Med. (2023) 31(2):52.

[42] Moodley D Esterhuizen TM Pather T Chetty V Ngaleka L. High HIV incidence during pregnancy: compelling reason for repeat HIV testing. Aids. (2009) 23(10):1255–9. 10.1097/QAD.0b013e32832a5934 19455017

[43] Thomson KA Hughes J Baeten JM John-Stewart G Celum C Cohen CR Increased risk of HIV acquisition among women throughout pregnancy and during the postpartum period: a prospective per-coital-act analysis among women with HIV-infected partners. J Infect Dis. (2018) 218(1):16–25. 10.1093/infdis/jiy113 29514254 PMC5989601

[44] Brubaker SG Bukusi EA Odoyo J Achando J Okumu A Cohen CR. Pregnancy and HIV transmission among HIV-discordant couples in a clinical trial in Kisumu, Kenya. HIV Med. (2011) 12(5):316–21. 10.1111/j.1468-1293.2010.00884.x 21205129

[45] Tang H Wu Z Mao Y Cepeda J Morano J. Risk factor associated with negative spouse HIV seroconversion among sero-different couples: a nested case-control retrospective survey study in 30 counties in rural China. PLoS One. (2016) 11(10):e0164761. 10.1371/journal.pone.0164761 27741292 PMC5065194

[46] Gray RH Li X Kigozi G Serwadda D Brahmbhatt H Wabwire-Mangen F Increased risk of incident HIV during pregnancy in Rakai, Uganda: a prospective study. Lancet. (2005) 366(9492):1182–8. 10.1016/S0140-6736(05)67481-8 16198767

[47] Keating MA Hamela G Miller WC Moses A Hoffman IF Hosseinipour MC. High HIV incidence and sexual behavior change among pregnant women in Lilongwe, Malawi: implications for the risk of HIV acquisition. PLoS One. (2012) 7(6):e39109. 10.1371/journal.pone.0039109 22768063 PMC3387180

[48] Karim SSA Richardson BA Ramjee G Hoffman IF Chirenje ZM Taha T Safety and effectiveness of BufferGel and 0.5% PRO2000 gel for the prevention of HIV infection in women. Aids. (2011) 25(7):957–66. 10.1097/QAD.0b013e32834541d9 21330907 PMC3083640

[49] Mugo NR Heffron R Donnell D Wald A Were EO Rees H Increased risk of HIV-1 transmission in pregnancy: a prospective study among African HIV-1-serodiscordant couples. Aids. (2011) 25(15):1887–95. 10.1097/QAD.0b013e32834a9338 21785321 PMC3173565

[50] Groer M El-Badri N Djeu J Harrington M Van Eepoel J. Suppression of natural killer cell cytotoxicity in postpartum women. Am J Reprod Immunol. (2010) 63(3):209–13. 10.1111/j.1600-0897.2009.00788.x 20055786 PMC2861128

[51] Hapgood JP Kaushic C Hel Z. Hormonal contraception and HIV-1 acquisition: biological mechanisms. Endocr Rev. (2018) 39(1):36–78. 10.1210/er.2017-00103 29309550 PMC5807094

[52] Drake AL Wagner A Richardson B John-Stewart G. Incident HIV during pregnancy and postpartum and risk of mother-to-child HIV transmission: a systematic review and meta-analysis. PLoS Med. (2014) 11(2):e1001608. 10.1371/journal.pmed.1001608 24586123 PMC3934828

[53] Birkhead GS Pulver WP Warren BL Hackel S Rodríguez D Smith L. Acquiring human immunodeficiency virus during pregnancy and mother-to-child transmission in New York: 2002–2006. Obstet Gynecol. (2010) 115(6):1247–55. 10.1097/AOG.0b013e3181e00955 20502297

[54] Lu C Jia Y Chen L Ding Y Yang J Chen M Pharmacokinetics and food interaction of a novel prodrug of tenofovir, tenofovir dipivoxil fumarate, in healthy volunteers. J Clin Pharm Ther. (2013) 38(2):136–40. 10.1111/jcpt.12023 23278367

[55] Lamorde M Byakika-Kibwika P Tamale WS Kiweewa F Ryan M Amara A Effect of food on the steady-state pharmacokinetics of tenofovir and emtricitabine plus efavirenz in Ugandan adults. AIDS Res Treat. (2012) 2012:105980. 10.1155/2012/105980 22454762 PMC3290802

[56] Valade E Tréluyer J-M Illamola SM Bouazza N Foissac F De Sousa Mendes M Emtricitabine seminal plasma and blood plasma population pharmacokinetics in HIV-infected men in the EVARIST ANRS-EP 49 study. Antimicrob Agents Chemother. (2015) 59(11):6800–6. 10.1128/AAC.01517-15 26282407 PMC4604421

[57] Eke AC Shoji K Best BM Momper JD Stek AM Cressey TR Population pharmacokinetics of tenofovir in pregnant and postpartum women using tenofovir disoproxil fumarate. Antimicrob Agents Chemother. (2021) 65(3):10–128. 10.1128/AAC.02168-20 PMC8092509 33318014

[58] Garcia-Cremades M Vučićević K Hendrix CW Jayachandran P Jarlsberg L Grant R Characterizing HIV-preventive, plasma tenofovir concentrations—a pooled participant-level data analysis from human immunodeficiency virus preexposure prophylaxis clinical trials. Clin Infect Dis. (2022) 75(11):1873–82. 10.1093/cid/ciac313 35474481 PMC10139701

